# What is the temperature of a moving body?

**DOI:** 10.1038/s41598-017-17526-4

**Published:** 2017-12-15

**Authors:** Cristian Farías, Victor A. Pinto, Pablo S. Moya

**Affiliations:** 1 Departamento de Ciencias Matemáticas y Físicas, Universidad Católica de Temuco, Temuco, Chile; 2 Department of Atmospheric and Oceanic Sciences, University of California, Los Angeles, Los Angeles, CA USA; 3 Departamento de Física, Facultad de Ciencias, Universidad de Chile, Santiago, Chile

## Abstract

The construction of a relativistic thermodynamics theory is still controversial after more than 110 years. To the date there is no agreement on which set of relativistic transformations of thermodynamic quantities is the correct one, or if the problem even has a solution. Starting from Planck and Einstein, several authors have proposed their own reasoning, concluding that a moving body could appear cooler, hotter or at the same temperature as measured by a local observer. In this article we present a review of the main theories of relativistic thermodynamics, with an special emphasis on the physical assumptions adopted by each one. We also present a set of relativistic transformations that we have derived by assuming the laws of Thermodynamics to be covariant. We found that under such assumptions a moving body appears to be hotter. Since relativistic thermodynamics is a topic that can be treated as part of an undergraduate course of classical thermodynamics or modern physics, the review and our own derivations presented here aim to encourage undergraduate physics students to open a discussion on the fundamental assumptions in thermodynamics and to engage in research activities early in their scientific career.

## Introduction

Constructing a theory of thermodynamics that is consistent with the principles of special relativity has been a long standing problem that can be traced back more than a hundred years ago. Shortly after Einstein started a revolution in physics with his famous work on special relativity, a problem was proposed: consider a system *A* in thermodynamic equilibrium, and two inertial frames, *I* and *I*′. Frame *I* is at rest with respect to *A* and *I*′ is moving with speed **w** with respect to *I*. Is it possible to find a relativistic transformation to express the thermodynamic quantities (such as temperature, pressure, heat transfer, entropy, etc.) of *A* in the system *I*′?

While the question is relatively simple, the answer is still up for discussion and the problem itself has led to a long-standing controversy in physics. Early attempts to solve the problem were proposed by Planck^1,2^ and Einstein^3,4^. They both proposed the same set of relativistic transformations:

1 $$\begin{array}{ccc}T^{\prime} =\frac{T}{\gamma }\,, & S^{\prime} =S, & p^{\prime} =p\,,\end{array}$$

where *γ* = (1 − (*w*/*c*))^−1/2^ is the Lorentz factor, *c* is the speed of light, and primed quantities correspond to the thermodynamic measurements in *I*’. These results mean that a body should appear cooler for a moving observer, but both entropy and pressure are relativistic invariants. This set of transformations was accepted by the scientific community for almost 50 years, however, Ott^5^ revisited the subject and suggested a different approach that resulted in another temperature transformation. Ott’s set of transformations are:

2 $$\begin{array}{ccc}T^{\prime} =\gamma T, & S^{\prime} =S, & p^{\prime} =p.\end{array}$$

These transformations mean that a moving body appears hotter, the opposite of what Einstein and Planck had suggested more than 50 years before.

Ott’s view on the temperature transformation gained support from other authors in the years that followed his first article^6,7^, but the controversy around who was right was only starting. Just a few years later, Landsberg^8^ proposed that the temperature should be a Lorentz invariant. His set of transformations are:

3 $$\,\begin{array}{ccc}T^{\prime} =T, & S^{\prime} =S, & p^{\prime} =p.\end{array}$$

These three main views led to a number of articles each one supporting one view or another. Three years after Landsberg, a fourth point of view was proposed by Cavalleri and Salgarelli^9^. They suggested that it only makes sense to define temperature in the rest frame *I*, and therefore, their conclusion is that no general Lorentz transformation for temperature and other thermodynamic quantities exist. This last view has also been supported by an important number of authors^10–13^. It is interesting to note that Einstein himself supported each one of these four theories at the latter stages of his life^14^.

As the focus of research has been to obtain a set of Lorentz transformations for thermodynamic quantities by using thermodynamical arguments, followed by the application of physical and mathematical tools proper of special relativity, most of the controversy comes from the initial assumptions and focus on the basic definitions of thermodynamic quantities chosen by each author, (e.g. temperature and heat transfer). Because of the many choices available and the difficulty to test the hypothesis with real experiments, the problem remains open, as to this day there is no agreement on which set of relativistic transformation of the thermodynamic quantities is correct, and why.

The problem itself is of easy formulation and very controversial in its resolution, therefore we think it is interesting as a teaching tool for students at the undergraduate level as they have the required level to understand the concept, and at the same time can greatly benefit by learning what science is at its core: an open discussion for different theories and ideas, where the focus are the fundamental ideas, definitions, and assumptions. Engaging the students in science discussions has been proved helpful in the development of critical thinking and communication skills^15^, which is important in the formation of future scientists^16^ and desirable for any professional.

This article is organized as follows: In section 2 we present a detailed review of the main ideas that have been proposed in relativistic thermodynamics. In section 3 we discuss an alternative formulation of relativistic thermodynamics, which is based on the treatment of Callen^17^, and that assumes that all the Laws of Thermodynamics are covariant. We focus the discussion in terms of the main thermodynamic definitions of each relevant physical quantity (e.g. temperature, pressure). Finally, we present our main takeaways in section 4.

## Transformations of Thermodynamic Quantities

### Planck-Einstein Transformations

In 1907 Einstein published the first theory of relativistic thermodynamics^3^. It considered a physical system surrounded by a case that was impenetrable to radiation. Einstein argued that, since the thermodynamic system is at rest in the *I*′ frame and moving with speed $w\hat{x}$ with respect to *I*, the Cartesian components of a given force **K** transform as

4 $$\begin{array}{ccc}{K}_{x^{\prime} }={K}_{x}\,, & {K}_{y^{\prime} }={K}_{y}/\gamma , & {K}_{z^{\prime} }={K}_{z}/\gamma .\end{array}$$

Then, if the forces are pressure-produced, they can be written as

5 $$\begin{array}{ccc}{K}_{x^{\prime} }=p^{\prime} {s^{\prime} }_{x}, & {K^{\prime} }_{y}=p^{\prime} {s^{\prime} }_{y}, & {K^{\prime} }_{z}=p^{\prime} {s^{\prime} }_{z}\,,\end{array}$$

where $\hat{n}\text{'}=({s^{\prime} }_{x},{s^{\prime} }_{y},{s^{\prime} }_{z})$ is the vector normal to the surface where the pressure is applied, and is pointing towards the body’s interior. He also considered transformations of the components $\hat{n}\text{'}$ as

6 $$\begin{array}{ccc}{s}_{x^{\prime} }={s}_{x}, & {s}_{y^{\prime} }={s}_{y}/\gamma , & {s}_{z^{\prime} }={s}_{z}/\gamma ,\end{array}$$

and then the forces in the *I* frame are

7 $$\begin{array}{ccc}{K}_{x}=p^{\prime} {s}_{x}, & {K}_{y}=p^{\prime} {s}_{y}, & {K}_{z}=p^{\prime} {s}_{z},\end{array}$$

from where is concluded that the pressure is a Lorentz invariant. Namely,

8 $$p^{\prime} =p\,.$$

Einstein repeated Planck’s argument that entropy does not depend on the choice of the reference system. In his argument, Planck^1^ considered a thought experiment in which a physical system *A* is moved by a reversible and adiabatic process from being at rest with respect to the *I* frame to being at rest with respect to a moving frame *I*′. Due to the reversibility of such a process, entropy does not change, and its value must be the same in both reference frames. i.e., entropy is a Lorentz invariant^1^. Einstein also proposed that in the *I*′ frame the heat transfer *dQ*′ can be written as an exact differential as

9 $$dQ^{\prime} =dE^{\prime} +p^{\prime} dV^{\prime} -{\bf{w}}\cdot d{\bf{G}}\text{'},$$

where *E* is the total energy of the system, not just the internal energy *U*, and **G** is the momentum vector. Then, using the set of transformations

10 $$E^{\prime} =\gamma [m{c}^{2}+E+{(\frac{w}{c})}^{2}pV]\,,$$

11 $${\bf{G}}=\gamma [m+(\frac{E+pV}{{c}^{2}})]\cdot {\bf{w}}\,,$$

along with the standard Lorentz transformation of a volume *V*, and using Eq. (8),

12 $$dE^{\prime} =\gamma [dE+{(\frac{w}{c})}^{2}(Vdp+pdV)]\,,$$

13 $$d{\bf{G}}=\gamma \frac{{\bf{w}}}{{c}^{2}}(dE+pdV+vdP\mathrm{))}.$$

Using these results on Eq. (9), he obtained the heat transfer transformation as

14 $$dQ^{\prime} =dQ/\gamma \,.$$

Then, assuming that the thermodynamic relationship between heat transfer, temperature and entropy change given by

15 $$dQ=T\,dS$$

is valid in all inertial frames for a reversible process, Eqs (14) and (15) can be combined to derive a temperature transformation given by

16 $$T^{\prime} =\frac{T}{\gamma }\,,$$

which means that a moving body appears cooler for a non local observer. This temperature transformation was strongly supported by Planck^2^ and accepted by several authors^18–20^ for more than fifty years.

### Ott: half a century later

In 1963, Ott revisited the problem, but started from the assumption that heat transfer should transform as an energy, meaning that

17 $$dQ^{\prime} =\gamma dQ,$$

the opposite of Eq. (14)^5^. He also considered that entropy should be a Lorentz invariant, and that Eq. (15) holds true for all inertial frames, and with those assumptions, derived the following temperature transformation:

18 $$T^{\prime} =\gamma T.$$

Ott’s result means that a moving body appears hotter, opposite to the Einstein-Planck temperature transformation. However, Ott adhered to Einstein’s argument on the invariance of pressure [see Eq. (8)]. His results were supported by Arzeliès, who also formulated the problem using four-vectors^6,21^ and reached the same conclusion. This transformation was supported by a number of authors^7,22^. In particular, Sutcliffe^7^ noted that from the Ott-Arzeliès transformations can be concluded that the expressions for the equation of state of an ideal gas and the variation of entropy in the Ott-Arzeliès theory

19 $$\begin{array}{cc}{\gamma }^{2}pV=nRT & dS=\frac{1}{T}(dU+{\gamma }^{2}pdV),\end{array}$$

are not covariant. This result is in contradiction with Einstein’s arguments that the mathematical expressions for the first and second law of thermodynamics have to be covariant. Additionally, the equation of state of an ideal gas does not have the same form in all reference frames. This is because the derivation of this particular equation takes into consideration the average of velocities of a high number of particles without inter-atomic forces, and thus does not depend on the velocity of the center of mass of the system.

Sutcliffe^7^ also used the statistical definition of entropy, which is a measure of the number of possible microscopic states of a given system, involving the probabilities *P*_*i*_ of the system to be in a microstate *N*_*i*_. Under this definition, entropy must be Lorentz invariant. Later on, he assumed that the classical thermodynamic definitions for temperature and pressure given by

20 $$\begin{array}{cc}T={(\frac{\partial U}{\partial S})}_{V} & p=-{(\frac{\partial U}{\partial S})}_{S}\end{array}$$

are valid in all reference frames. He found the result given by (18), and also a different transformation for pressure, given by

21 $$p^{\prime} ={\gamma }^{2}p.$$

Sutcliffe noticed that this transformation is crucial to ensure that the equation of state of and ideal gas, and the entropy variation

22 $$\begin{array}{cc}pV=nRT\, & dS=\frac{1}{T}(dU+pdV),\end{array}$$

are covariant; i.e. both expressions hold for all reference frames. To explain the discrepancy between his pressure transformation [see Eq. (21)] and the one by Ott^5^ and Arzelies^6^ [see Eq. (20)], he argued that the definition of pressure given by Eq. (20) is the *thermodynamic pressure*, whilst the one used by previous authors (e.g^1,3,5,6^.) was the so called *mechanical pressure*.

Of note, Einstein, while revisiting the issue of relativistic thermodynamics in his correspondence with Von Laue in 1952, concluded that the correct temperature transformation should be the one proposed in Ott-Arzeliès theory^14^. His argument to support Ott’s transformation consisted on the analysis of the heat exchange between two heat reservoirs, *L* and *L*′, both having the same rest temperature *T*. One of the two reservoirs, (*L*′), was moved by a “machine” in an adiabatic and reversible process, with the “machine” being an auxiliary reservoir, also with temperature *T*, which is at rest respect to *L*′. Einstein considered that there was a cyclic process where *L* transfers an amount of heat Δ*Q* to the machine, which is moving with a velocity **w**. Then the machine releases an amount of heat Δ*Q*′ to *L*′, recovering its initial rest-heat. Since heat transfer is an energy, Einstein argued that Δ*Q*′ = *γ*Δ*Q*. Then, the amount of mechanical work done during this process was

23 $${\rm{\Delta }}W={\rm{\Delta }}Q^{\prime} -{\rm{\Delta }}Q={\rm{\Delta }}Q(\gamma -\mathrm{1)}\,,$$

from where he concluded that the temperature transformation was the one given by Eq. (18). Einstein’s conclusions came over a decade before the publication of the original article by Ott, but his results were not published.

### Temperature as an invariant

The existence of two opposite solutions to the same problem created a controversy on the correct temperature transformation that led to a significant number of publications during the decades of 1960 and 1970^7,9,10,22–26^. Among those studies a new interesting theory appeared on 1966 and 1967 when Landsberg published two articles questioning the results of Einstein and Planck on the temperature transformation, and proposing that the temperature is a Lorentz invariant^8,25^. He pointed out to unconvincing physical implications with the generalization of the thermodynamic definition of temperature given by Einstein, which is written as

24 $$\frac{1}{T}={(\frac{\partial S}{\partial E})}_{V,P}$$

in all inertial reference frames. Here, *E* is the total energy of the physical system, and not only his internal energy *U*. Landsberg stated that, while this definition is mathematically correct, it contradicts the statistical definition of temperature, which comes from the study of the relative motion of a large number of particles with respect to their center of mass. Therefore, all statistical definitions can not depend on the velocity of the center of mass of the system, and must remain invariant^8^. This is

25 $$T^{\prime} =T.$$

Landsberg then proposed a new definition of temperature that ensures relativistic invariance and a different generalization of the definition of temperature in relativistic thermodynamics given by

26 $$\frac{1}{T}=\frac{1}{\gamma }\,{(\frac{\partial S}{\partial E})}_{V,P}.$$

This result imply that the internal energy is a Lorentz invariant, and since *TdS* is also an invariant, then we can define the temperature as

27 $$\frac{1}{T}={(\frac{\partial S}{\partial U})}_{V,P}$$

in all frames of reference. This view was supported by a number of authors (see for example refs^23,27^), all of which put special emphasis in the definition of temperature. Cavalleri and Salgarelli^9^ in particular stated that temperature is a concept that only makes sense if it can be locally measured. Since this can only happen on the rest frame of the physical system, then temperature has to be Lorentz invariant. This is in agreement with the statistical definition of temperature.

It is also very interesting to note that Einstein, in his correspondence with von Laue in 1952–1953, once again changed his mind on the issue of the temperature transformation, this time supporting the notion of considering temperature as a Lorentz invariant^14,28^. Unfortunately, these results were never published, and we know them now only because the disclosure of his personal correspondence with von Laue^28^.

### No general temperature transformation

Not long after Landsberg proposed that temperature is Lorentz invariant, he focused on the the problem of defining temperature, first by noticing issues with a kinematic definition in the scope of special relativity^25^, and later by studying its implications on the obtention of the “right” relativistic transformation^29^. He also designed a thought experiment which would serve to find the right relativistic temperature transformation, understanding that it is only through experimental observations that a theoretical controversy can be settled^30^. Nevertheless, he did acknowledge that the experimental difficulties of this design may be prohibitive, but concluded that a temperature transformation must exist. Later, Krizan studied the possibility that observations in microwave radiation can provide a way to decide which temperature transformation is the right one between the proposals of Einstein^3^, Ott^5^ and Landsberg^8^. However, he concluded that this is impossible because these transformations violate the first and zeroth law of thermodynamics in a statistical distribution of black body radiation^24^. More recently, Kaniadakis mentioned that cosmic rays observations may be useful to test different relativistic theories, and therefore could be relevant to study in the future^31,32^.

Given the impossibility to obtain experimental evidence supporting a particular theory of relativistic transformations of the thermodynamic quantities, the majority of the discussion has been based on theoretical arguments and thought experiments. After the developments of Einstein^3^, Ott^5^ and Landsberg^8^ several authors began to study the basic assumptions of the different relativistic thermodynamics theories, in order to determine which one is correct. Cavalleri and Salgarelli^9^ and Newburgh^10^ noticed that different definitions of temperature lead to different relativistic transformations of it. In particular, Newburgh differentiates between a *kinematic* and a *dynamic* problem. In the kinematic problem, the physical system is at rest with a reference frame *I*, which also contains all the instruments measuring its thermodynamic quantities. The second frame, *I*′, is moving with a velocity ***w***. Then the question is how the observer of *I*′ measures the thermodynamic quantities of the physical system in *I*. Both observers *I* and *I*′ take measurements of the same physical system, without changing its properties in the process. On the other hand, in the dynamical problem, the physical system is originally at rest respect to a reference frame *I*′, and then starts to move. The question is how the observer in *I*′ measures the thermodynamic quantities of the physical system while the system is in motion. The problem here is that setting the system in motion changes its thermodynamic state, and therefore each different mechanism that set the thermodynamic body in motion will lead to a different relativistic transformation of its thermodynamic quantities. Newburgh showed that the relativistic invariance of radiated power in a purely kinematic problem leads to the Ott temperature transformation^10^.

Since Newburgh^10^, several authors came to the conclusion that different definitions of a thermometer lead to different temperature transformations, and hence all these works have supported the position that it is not possible to find a general relativistic transformation for temperature^11–13,33^. In particular, Landsberg and Matsas concluded that an observer that is moving inside a heat reservoir cannot detect a black body radiation pattern, and therefore can not find a parameter which can be identified as temperature^11^. Later, Montakhab *et al*.^12^ carried out an analysis of a two dimensional relativistic gas using molecular dynamics simulations and concluded that, while thermal equilibrium can be detected in both *I* and *I*′ reference frames, statistical methods to define temperature can not provide an answer of which transformation is the right one for temperature. Moreover, Bíró and Ván^13^ studied the energy-momentum density of a one-component fluid in order to obtain the temperature transformation. They argued that relativistic thermodynamics can be obtained by integrating the local energy-momentum conservation on an extended and homogeneous thermodynamic body. They obtained each one of the transformations given by Eqs (16), (18), and (25) after assuming different mechanisms on internal heat transfer. In addition, Nakamura^13^ obtained a similar result as Bíró and Ván^13^. He started from the covariant formalism of relativistic thermodynamics proposed by VanKampen^26^ and concluded that the three transformations presented in Eqs (16), (18) and (25) are correct depending on the initial assumptions of heat transfer and three-dimensional volume transformations^33^.

In recent years, and with the controversy over relativistic transformations for a thermodynamical system still open after more than a century, new tools and computational capabilities have led a to a series of more complex numerical experiments and solutions. For example, the use of molecular dynamics simulations to obtain a statistical definition of temperature^34,35^ and expansions on covariant formalism for relativistic thermodynamics^36,37^ have been proposed. In the case of the simulations, Cubero *et al*.^34^ Liu^35^ proposed a Jüttner velocity distribution^38^ as the correct one for a relativistic gas, but each one of them found different temperature transformations, supporting Landsberg^34^ and Planck-Einstein^35^ results. On the other hand, Requardt^36^ and later Przanowski^37^ considered covariant formulations of relativistic thermodynamics, and found that the most likely temperature transformation is the one proposed by Ott. Interestingly, Przanowski and Tosiek^37^ uses the same argument of Einstein mentioned at the end of subsection 2.2 to support that a moving body appears hotter, but also acknowledging that different definitions of a “statistical thermometer” lead to different temperature transformations. Recently, Dunkel *et al*.^39^ pointed out that the main reason for the existence of the controversy is the number of different definitions of heat and work, all equally plausible, that lead to different conclusions and transformations of the thermodynamic quantities. In their study they defined the thermodynamic quantities with respect to the backward-lightcone of an observation event, and showed that it is possible to obtain both Planck-Einstein^1,3^ and Ott^5^ formalisms by taking different choices in their definitions. Dunkel *et al*.^39^ also suggested that, while Ott (and later Van Kampen) results seem to be more reasonable, it is almost impossible to tell which one is the correct one, given the current impossibility to perform an experiment which could shed light into this topic. Within this context, we then conclude that the long-standing controversy on the construction of a theory of relativistic thermodynamics is mainly based on the initial assumptions, which need to be tested in the future in order to discern which set of Lorentz transformations is correct for quantities such as temperature and pressure.

### A simpler approach to relativistic thermodynamics

In order to focus on the root of the problem at a level appropriate for undergrad students, we want to suggest a different and simpler approach. We use basic thermodynamic definitions and equations to focus on the discussion of the merits of such assumptions rather than on the final result. We use the treatment of Callen^17^, which is closer to Statistical Mechanics, and is standard in a Thermodynamics course at undergraduate level. Therefore, we will focus on compound isolated systems that exchange extensive thermodynamic quantities in order to define the intensive quantities. In a classic, non-relativistic case, this means that quantities such as temperature and pressure can be defined when two isolated subsystems are in thermodynamic equilibrium, after they have exchanged a certain extensive quantity (such as heat or volume). When this happens, we can identify an intensive variable as the parameter that is equal in both subsystems when they reached equilibrium. Thus, temperature is equal in both subsystems when they exchange heat (but no volume or particles) through a fixed diathermic and impermeable wall, pressure is the same in both subsystems when they exchange volume (but no heat or particles) through a movable adiabatic wall, and the chemical potential is equal in both subsystems when they exchange particles (but no heat or volume) through a permeable fixed and adiabatic wall. Callen^17^ states that the difference in the internal energy *dU* of any subsystem can be written as

28 $$dU=TdS-pdV+\mu dN,$$

where the extensive quantities *U*, *S*, *V*, and *N* are the internal energy, entropy, volume, and number of particles of the subsystem. The intensive quantities *T*, *p*, and *μ* are the temperature, pressure, and chemical potential, respectively. From Eq. (28) the variation of entropy in the same system can be formulated as

29 $$dS=\frac{1}{T}dU+\frac{p}{T}dV-\frac{\mu }{T}dN.$$

Since *dS* given by (29) is a complete differential, Callen defines 1/*T*, *p*/*T* and *μ*/*T* as

30 $$\begin{array}{ccc}\frac{1}{T}={(\frac{\partial S}{\partial U})}_{V,N}, & \frac{p}{T}={(\frac{\partial S}{\partial V})}_{U,N}, & \frac{\mu }{T}=-{(\frac{\partial S}{\partial N})}_{U,V}.\end{array}\,$$

Callen also showed that, when there is thermodynamic equilibrium between two subsystems after a process, these formal mathematical definitions correspond to the thermodynamic definitions of the intensive quantities. Therefore, it is mandatory to have thermodynamic equilibrium between subsystems in this treatment.

When we have relative motion between two subsystems, we need to extend the classic definitions by Callen^17^. This means that we need to define a way in which both subsystems interact, so all the intensive quantities are defined by the means of a proper thermodynamic process after the systems reach equilibrium. This is possible since macroscopic thermodynamic equilibrium can be achieved even when two systems are in relative motion. However, in such case, besides *U*, *V*, and *N*, the total momentum of the system ***P*** has to be taken into account as another relevant extensive quantity (see for example Diu *et al*.^40^).

Let us consider that the thermodynamic system under study *A*, which is at rest in the *I* frame, interacts with a second subsystem *A*′ (at rest in the *I*′ frame) by the means of a bath to which both systems are connected. For example, if we have a thermal bath, the system *A* can exchange heat (but no particles, work, or another extensive quantity) with it. Then, the heat reservoir will go back to its initial state by transferring the extra heat to the subsystem *A*′. This will allow us to define a temperature scale in both frames *I* and *I*′. This type of process also occurs for exchange of particles and volume (hence work), but we can also allow the exchange of momentum between the subsystems. This means that the entropy variation of the system *A* during a thermodynamic process, given by Eq. (29) needs to be extended so we can use it in all inertial frames, considering this momentum exchange. Mathematically we can see that if the total rest mass of our thermodynamical system is *M*, then its total energy must consider the sum of the kinetic and rest energy *E*_*m*_, namely

31 $${E}_{m}^{2}={{\bf{P}}}^{2}{c}^{2}+{M}^{2}{c}^{4},$$

where ***P*** is the momentum of the system, given by **P** = *γM* **w**. Then, the energy variation *dE*_*m*_ in *A* after it exchanges momentum with another subsystem (but no internal energy, volume, and particles) is

32 $$d{E}_{m}=\frac{{\bf{P}}}{{({{\bf{P}}}^{2}{c}^{2}+{M}^{2}{c}^{4})}^{\mathrm{1/2}}}\cdot d{\bf{P}}.$$

Considering that *γ* = (1 + (*p*/*Mc*)^2^)^1/2^ and ***P*** = *γM* **w**, we can write

33 $$d{E}_{m}={\bf{w}}\cdot d{\bf{P}}.$$

Note that in Eq. (33) we can formally identify the velocity **w** as the intensive quantity that is equal in two subsystems when they have stopped their exchange of momentum. Then, we can write the total variation of energy as

34 $$dE=dU+d{E}_{m},$$

where *E* is the total energy of the system. Therefore, *dE* is given by

35 $$dE=TdS-pdV+\mu dN+{\bf{w}}\cdot d{\bf{P}},$$

from where we can write the entropy element *dS* as

36 $$dS=\frac{1}{T}dE+\frac{p}{T}dV-\frac{\mu }{T}dN-\frac{{\bf{w}}}{T}\cdot d{\bf{P}}.$$

It is important to note that, even when the variation of entropy in the system *A* now depends on the variation of momentum, entropy itself is Lorentz invariant due to its statistical definition.

While the expression of *dE* and *dS* given by Eqs (34) and (36) are formally correct, they need to have a proper thermodynamic meaning now that they have an extra term. In that regard, we can think that the systems *A*′ and *A* exchange kinetic energy (but not heat, particles, or volume) as the result of an adiabatic transfer of momentum from *A* to a momentum bath, which in turn transfers kinetic energy to *A*′. Then, in the *I*′ frame, we can interpret the intensive variable linked to the transfer of momentum as the velocity of displacement between frames. Note that Eq. (36) is a complete differential, from where we can mathematically define 1/*T* and *p*/*T* as

37 $$\begin{array}{cc}\frac{1}{T}={(\frac{\partial S}{\partial E})}_{V,N,{\bf{P}}}, & \frac{p}{T}=-{(\frac{\partial S}{\partial V})}_{E,N,{\bf{P}}},\end{array}$$

mathematical expressions that have a proper thermodynamic meaning as they arise from the analysis of a thermodynamic process that involves the exchange of energy between two isolated systems.

For the relativistic case, we consider that the system *A* exchanges heat (but no particles, volume, or momentum) with a heat reservoir, reaching thermodynamic equilibrium. As already mentioned, this happens when the intensive quantity identified as temperature is the same in both subsystems (system *A* and the reservoir). After the exchange of heat there is a change in the internal energy of *A* given by *dE* = *dU* = *dQ* = *TdS*. Then, since *E*_*m*_ = 0 in the *I* frame, we have ∂*S*/∂*E*|_*V*,*N*,***P***_ = ∂*S*/∂*U*|_*V*,*N*,***P***_ = 1/*T* as a temperature scale in thermodynamic equilibrium. The *A*′ system, at rest in the *I*′ frame, is also connected to the heat reservoir, which needs to get back to its initial state. Therefore, there is a change of energy *dE*′ from the reservoir to the *A*′ system, which means that we can write *dE*′ = *dQ*′ = *T*′*dS*′ in the *I*′ frame. Since *A*′ is also in equilibrium with the heat reservoir, this allows us to identify

38 $$\frac{1}{T^{\prime} }={(\frac{\partial S^{\prime} }{\partial E^{\prime} })}_{V^{\prime} ,N^{\prime} ,{\bf{P}}\text{'}},$$

as a temperature scale in *I*′. Note that we have defined temperature considering the total energy of a system instead of just the internal energy. This is because we identify this intensive quantity as the one that is equal between two subsystems in equilibrium when we allow only the exchange of heat. One consequence of this is that we have a *thermodynamic* thermometer, which measures a temperature that depends not only on the internal interactions between the particles that compose the physical system *A*, but that also contains the motion between inertial frames. Therefore, it makes sense to talk about an *apparent* temperature instead of a *rest* temperature in this approach. The rest temperature of a body, in accordance with statistical mechanics, depends on the relative motion of all the composing particles respect to the center of mass of the system, and it should be Lorentz invariant.

We can define pressure in a relativistic environment following a similar approach. We consider the systems *A* and *A*′ to be connected to a reservoir, and we allow only exchanges of volume (hence work), between *A* and the reservoir, and then between the reservoir and *A*′. After reaching equilibrium, the system *A* has transferred an amount of work *dW* = −*pdV* to the reservoir, which in turn transfer an amount of work *dW*′ = −*p*′*dV*′ to the system in the *I*′ frame. Therefore, we can identify pressure as the intensive quantity that is equal between two subsystems when they have reached thermodynamic equilibrium, just as is done in Callen’s approach^17^. This definition does not guarantee that the pressure has the same mechanical meaning of force divided by area in all reference frames. Therefore, in this context the definition of pressure given by Eq. (37) has a physical meaning in all reference frames.

Using the definitions for temperature and pressure given in Eq. (37), the expression for the element *dS* given by Eq. (36), and assuming that entropy is a Lorentz invariant, we obtain a relativistic transformation for temperature given by

39 $$T^{\prime} =\gamma T,$$

which is the same result proposed by Ott^5^ and Arzelies^6^, and means that a moving body appears hotter. We can get the same result by considering that the system *A* transfers an amount of heat *dQ* to the reservoir, which in turn transfers an amount of heat *dQ*′ = *γdQ* to *A*′. From here we have *T*′*dS*′ = *γTdS* and, since entropy is Lorentz invariant, we obtain *T*′ = *γT* as the comparison between the two temperature scales observed in the different frames for the same system *A*.

Using Eq. (39) and the invariance of entropy in the definition of pressure given by Eq. (37), we can also find the a transformation for pressure:

40 $$p^{\prime} ={\gamma }^{2}p,$$

which is the same transformation proposed by Sutcliffe^7^ for what he called “thermodynamic pressure”. We can again obtain this relativistic transformation for pressure by considering that *A* transfers an amount of work *dW* to the reservoir, which in turn transfers an amount of work *dW*′ = *γdW* to *A*′. Then, since no exchange of heat, particles, or momentum is allowed, we have *p*′*dV*′ = *γpdV*, from there we obtain *p*′ = *γ* ^2^ *p*. On the other hand, if we had followed Einstein’s procedure to calculate the pressure transformation in a mechanic way (assuming that *p* = *F*/*A*), then we would have concluded that this quantity is a Lorentz invariant. This discrepancy is related to the different definitions of pressure. In our approach, that identifies intensive quantities as the ones that are equal between two subsystems in thermodynamic equilibrium, the set of relativistic transformations we obtain for them is a consequence of a particular thermodynamic process where we find equilibrium by using a reservoir as an auxiliary system. In the case of pressure, it is only equal to force divided by area in the rest frame of the system *A*. All this shows that we need to rely on external arguments about the definition of each thermodynamic quantity in order to obtain a relativistic transformation for each one of them, regardless of the correctness of our mathematical treatment. Moreover, we can conclude that different definitions will lead to different transformations, in agreement with Nakamura^33^.

Our approach to the problem is mathematically simple, and it is focused on how to define the intensive thermodynamic variables in the context of special relativity. Nevertheless, it raises several questions that can be discussed in the classroom. A crucial issue here is related to the definition of intensive variables such as temperature and pressure. In Callen’s approach,(what we are using here), these variables only have meaning when there is a process between two systems in which they exchange an extensive quantity until they reach equilibrium. Therefore, the first important question is how possible is to have thermodynamic equilibrium between two systems when one of them is moving respect to the other? We propose that equilibrium is possible if both systems are connected to a reservoir, and Diu *et al*.^40^ argues that equilibrium is possible even when the two systems are exchanging a certain extensive quantity without the use of a third, auxiliary system. But if *A* and *A*′ interact via a reservoir, how can we define a temperature (or a pressure) scale? Should we find another set of relativistic transformations in that case? Here we find a limitation of our approach, because we need external arguments to answer these questions.

Another interesting question relates to the compatibility of thermodynamics and special relativity. In thermodynamics we are only concerned about the initial and final states of subsystems that reach macroscopic equilibrium. Since Lorentz transformations mix space and time, can we still find a stationary equilibrium state for *A* in all (inertial) reference frames? Our approach shows that relativity and thermodynamics are compatible, under the condition that thermodynamic equilibrium is possible between the *A*′ and *A* subsystems. Since the equilibrium configuration of a system *A* is reached when entropy is maximized, and entropy is a Lorentz invariant, it should be possible to find a stationary state for *A* in all frames. But we need to point out that, while our approach allow us to argue that this is possible when *A* and *A*′ are connected to a reservoir, this might not occur with other processes, and therefore we might not be able to find a thermodynamic definition of temperature and pressure, for instance.

All the definitions of thermodynamic quantities reviewed in this article are based on elemental thermodynamics and statistical mechanics. The main source of the discussion is based on the definitions rather than on mathematical treatment. Therefore, the study of the controversies that surround relativistic thermodynamics has an enormous benefit potential to undergraduate students. They will be able to engage in discussions that are often not present in the classroom, where most of the teaching process has traditionally been based on the transmission of basic concepts that students have to use to solve problems^41,42^. Since only basic knowledge in thermodynamics and special relativity is enough to understand most of the treatments of each one of the views presented in this article, the study of relativistic thermodynamics can be explored by any second or third year student of an undergraduate career in Physics. We encourage professors to introduce it as part of the program in a Classical Thermodynamics course, or maybe as a side project in the class.

## Concluding Remarks

A review of the main views on a long-standing controversy in theoretical physics, which is the construction of a relativistic thermodynamics theory has been presented. We discussed the main physical ideas behind the different proposed transformations for thermodynamic quantities, with an emphasis on the temperature transformation, which has been the most controversial issue in the past, as four main different results have been proposed. We also looked at the current state of the discussion, and notice that this research topic is far from being closed. More ideas are needed, and in particular we need to obtain experimental evidence that can shed any light about the correct answer.

We have presented an alternative treatment of the problem, where by considering the variation of entropy *dS* as an exact differential, we obtained thermodynamic definitions for temperature and pressure, that led us to the set of transformations previously reported by Sutcliffe^7^. These transformations are mathematically correct, but are based on definitions whose physical validity can be disputed. The discussion around the definitions we propose here, and the definitions and choices reviewed in this article is of significant value within the context of a Classical Thermodynamics course. The professor in charge of the classroom can propose a wide range of views, engaging the students to do their own research, and pursue answers for a question that appears to be simple in its formulation, but that is still open. We concur with the view that in the current state of increasing professionalization of science, undergraduate students need the right incentives to pursue a career as a researcher, particularly at an early age. We believe that this is one of the research problems that can give students some of their firsts hints in the worklife of a researcher.
